# Retraction Note: Improved treatment of Asthma by using natural sources of antioxidants

**DOI:** 10.1186/2193-1801-3-558

**Published:** 2014-09-25

**Authors:** Nguyen Van Toan, Tran Thi Hanh

**Affiliations:** School of Biotechnology, International University, Ho Chi Minh City, Vietnam; Faculty of Applied Sciences, University of the West of England, Bristol, UK; Home Clinic, 345 D5 Street, Binh Thanh District, Ho Chi Minh City, Vietnam

## Retraction

The original version of this paper (Van Toan and Thi Hanh 2013) is retracted because of ethical concerns: the clinical trial was not approved by an ethical board and the authors did not provide evidence that patient consent was obtained. The scientific advisor for this clinical trial (ANZCTR 2012) at the University of the West of England UK (an affiliation of the corresponding author) indicated he was not aware of this study and that the university was not involved.

It is a requirement that experimental research reported in SpringerPlus was performed with the approval of an appropriate ethics committee, and that research carried out on humans must be in compliance with the guidelines of the World Medical Association.

Dr. Max Haring, Executive Editor for SpringerPlus
